# Component wave calculation and analysis of the acoustic field in a borehole within a three-phase porous medium

**DOI:** 10.1038/s41598-023-33709-8

**Published:** 2023-05-11

**Authors:** Lin Liu, Xiumei Zhang, Xiuming Wang, Xiao He, Hao Chen

**Affiliations:** 1 State Key Laboratory of Acoustics, Institute of Acoustics, Chinese Academy of Sciences, Beijing, 100190 China; 2 University of Chinese Academy of Sciences, Beijing, 100149 China; 3 Beijing Engineering Research Center for Drilling and Exploration, Institute of Acoustics, Chinese Academy of Sciences, Beijing, 100190 China

**Keywords:** Solid Earth sciences, Mathematics and computing, Physics

## Abstract

The knowledge of acoustic wave propagation in a borehole embedded in gas hydrate-bearing sediments is of great significance for the exploitation of gas hydrate. A gas hydrate-bearing sediment is a typical three-phase porous medium containing two solids and one fluid. However, until now, the borehole acoustic wavefield and its component waves within such a porous medium have never been calculated. In this work, a real-axis integration method is proposed to calculate the borehole acoustic field embedded in a three-phase porous medium based on the Biot-type three-phase theory. Meanwhile, a component wave approach, combined with the branch-cut integral method and the residue theorem, including residues at leaky poles is proposed to study the borehole wave propagation of a three-phase porous medium. The branch points and poles of the potential acoustic wave function are obtained, which correspond to the normal and leaky modes on various Riemann sheets. On this basis, the excitation intensity and waveforms of each component are obtained. The result shows that the waveform summed up from all individual waves agrees well with the full waveform calculated by real-axis integration, which provides a theoretical basis for the subsequent inversion of reservoir parameters by using the information of various mode waves.

## Introduction

Knowledge of the physical properties of the acoustic field in a borehole in a three-phase porous medium is essential for the exploitation of gas hydrate. Natural gas hydrates are recognized as a kind of potential energy source due to the huge amount of trapped gas and the role they play in global climate change and the carbon cycle, which abstracts attention^1–3^. In fact, natural gas hydrate-bearing sediments are typical three-phase porous media (solid grain, gas hydrate, and water), which implies a particular topological configuration, namely the one where solid grain and gas hydrate form two continuous and interpenetrating solid matrices^4–6^. Gas hydrates have a remarkable effect on wave velocities^7,8^. Hence, acoustic logging methods constitute the best way for quantifying the amount of gas hydrate. The compressional wave velocity is considered to be a significant property, providing information on gas hydrate saturation evaluation^9–12^. However, information on other waves obtained from acoustic logging is rarely used, because almost no profound research on wave propagation in a borehole surrounded by a three-phase porous medium has been reported until now.

Scholars have done extensive and in-depth theoretical research and simulation work on the acoustic field in a borehole by using the real-axis integration (RAI) method over the years. Biot^13^ first studied the propagation mechanism of borehole acoustic waves theoretically, provided the dispersion equation of guided waves (real mode wave) and calculated the dispersion curve of the guided waves of the hard formation, and concluded that a multiple mode wave with cut-off frequency (pseudo-Rayleigh wave) and Stoneley wave without cut-off frequency exist in a hard formation. White and Zechman^14^ gave the transient solution of the borehole acoustic field, and numerically simulated the theoretical full-wave for the first time, among which the head wave was displayed. Peterson^15^ first summarized critical refracted P-wave and critical refracted S-wave in a borehole as cut-line integrals through the branch points of P-wave and S-wave in the plane of complex wave number. Roever et al.^16^ used the ray expansion method to express the critical refraction wave described by cut-line integral as the superposition of multiple refractions. Rosenbaum^17^ first applied Biot’s theory^18,19^ to acoustic logging research, three different formations were selected to numerically study the influence of reservoir parameters such as porosity and permeability on the full wave in the borehole. It was found that the permeability was most closely related to the Stoneley wave. This is the beginning of the theoretical study of reservoir acoustic logging problems, however, he did not discuss the problem of wave separation. Component wave analysis is an important method to study the borehole acoustic field. Based on the Sommerfeld (SMF) secant and radiation condition, Kurkjian^20^ gave the univariate definition of the cut-line integral envelope for elastic medium, and compared the correspondence between P-wave head wave, S-wave calculated by different secant integral, guided wave and full wave. The calculation of full wave adopted the complex frequency method to exclude the influence of the singular point on the real axis. It is pointed out that the leaky mode of S-wave will affect the S-wave head wave, however, the correct numerical simulation results were not given. Peterson and Roever et al.^15,16^ consider the acoustic full wave field to be a sum of the pole and branch-cut contributions in the frequency-wavenumber domain. In general, each pole in this domain is associated with a mode wave, whereas each branch cut represents a head wave in the space–time domain^19^. Wang et al.^21^ and Zhang et al.^22^ studied the integral expression and component wave calculation of the acoustic field in a borehole embedded in an elastic formation, analyzed the structure of the Riemann surface of the characteristic function in a borehole, and proposed a method to represent and separate each component wave. Zhang et al.^23^ studied the numerical method to represent the component waves in a borehole and analyzed the distribution of poles that contribute to the borehole acoustic field and are located on different Riemann sheets. Zheng et al.^24^ obtained individual component waves in the acoustic logging-while-drilling environment using the branch-cut integral method and the residue theorem, including residues at leaky poles or sheets.

In the present paper, based on the Carcione–Leclaire three-phase theory, according to the equations of motion and constitutive relations, combined with the six constructed boundary conditions in three-phase porous media, a real axis integration (RAI) algorithm is proposed to obtain the borehole acoustic field in gas hydrate-bearing sediments (three-phase porous media). The structure of the Riemann surface of the characteristic function of the borehole in three-phase porous media is pointed out for the first time. On this basis, the single-valued definition of the characteristic function was brought forward and the integral contour was selected, the distribution of poles on the frequency-complex wave number domain is completely presented, based on which we can analyze the dispersion and the attenuation of the wave modes. To make better use of full-wave information and specifically analyze the characteristics of each mode, we get the excitation intensity of the corresponding wave mode by calculating the branch-cut integral and pole residue, thus getting the time-domain waveforms by Fast Fourier transform (FFT). In particular, the contribution of the Leaky mode I and the pseudo-Rayleigh wave to the S1 wave are investigated in detail. In addition, the influence of waveforms affected by the source center frequency and distance is discussed. Finally, the wave superposition and RAI results were compared to verify the effectiveness of the component wave calculation.

## Algorithm implementations

In this section, firstly, the borehole acoustic field excited by a point sound source in a three-phase porous formation is established, and the general solution of the Helmholtz equation in a cylindrical coordinate system is obtained by the method of separation of variables, which corresponds to the potential functions of the acoustic field in and out of the well, respectively. The coefficients of the acoustic field in the well are determined by solving the equations according to the boundary conditions at the side wall of the borehole.

The Carcione–Leclaire three-phase theory predicts three compressional and two shear waves exit when acoustic wave propagates in three-phase porous media: the first kind of compressional wave (P1), the first kind of shear (S1) wave, the second kind of compressional wave (P2), the second kind of shear (S2) wave and the third kind of compressional wave (P3). It is known that there are four boundary conditions used in solving the open hole problem in fluid-saturated porous media based on Biot’s theory. For a three-phase porous medium, besides the P3 wave is caused by the relative motion between solid grains and pore fluid, two elastic waves P2 and S2 are produced due to the existence of the solid in pores^5,25^. Therefore, the undetermined coefficients and boundary conditions are increased to six, which will be explained below.

**Figure 1:**
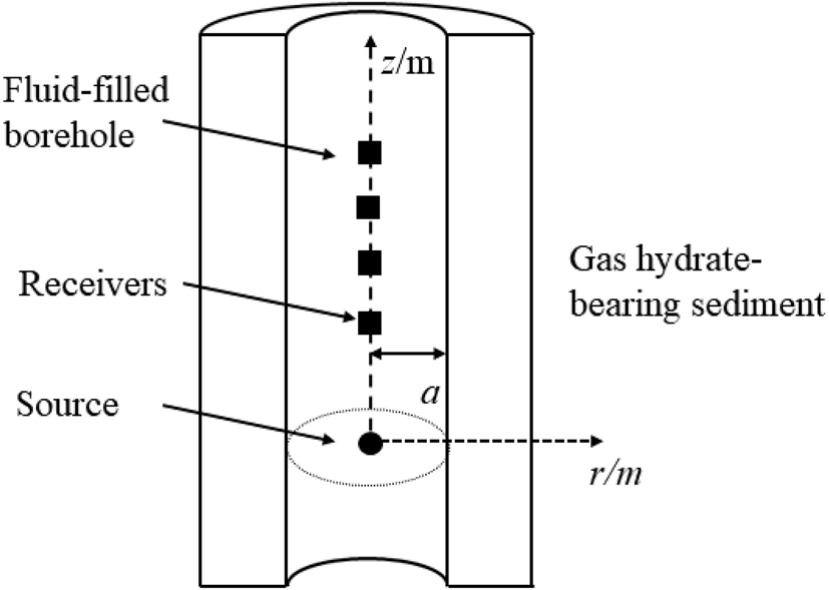
Schematic diagram of the acoustic logging model. The fluid-filled borehole is embedded in a gas hydrate formation. The borehole radius* a* is 0.1 m. The monopole acoustic source and the receivers are along the borehole axis, and the source is located at the origin of the cylindrical coordinate system.

We analyze the acoustic field in the borehole surrounded by a three-phase porous medium. The schematic of the acoustic logging model is presented in Fig. 1. A fluid-filled borehole is embedded in an unbounded three-phase porous formation (gas hydrate-bearing sediment). With a cylindrical coordinate system (*r*, *z*, *θ*), the borehole axis lies along the central axis *z* of the cylinder, the monopole acoustic source is located at the origin, and the source is at the center of the borehole, the squares represent the receivers. As the formation is axisymmetric, all field quantities are independent* θ*. The borehole radius *a* is 0.1 m, $\rho_{f}$ denotes the fluid (water) density, and $v_{f}$ represents the fluid (water) velocity. In the frequency-wavenumber domain, the acoustic field in the borehole^26^ can be expressed as

1 $$\varphi (k_{z} ,\;\omega ) = \frac{{{\text{i}}F}}{{\rho_{f} \omega^{2} }}\left[ {K_{0}^{{}} (\alpha_{f} r)} \right.\left. { + A_{f} (k_{z} ,\omega )I_{0} (\alpha_{f} r)} \right],$$

where *F* denotes the frequency spectrum of the source, $A_{f}$ is an undetermined coefficient, which can be regarded as the reflection coefficient of the borehole wall. In the above equation, $K_{0}$ and $I_{0}$ are the modified Bessel functions, and $i = \sqrt { - 1}$.

The acoustic field in a three-phase porous medium can be expressed by the displacements of solid grain frame $\vec{u}_{1}$, pore fluid $\vec{u}_{2}$ and gas hydrate $\vec{u}_{3}$. The three displacements produced by a monopole source^26^ can be written as

2 $$\begin{aligned} \vec{u}_{1} & = \nabla \varphi_{1} + \nabla \times \nabla \times (\eta_{1} \hat{z}), \\ \vec{u}_{2} & = \nabla \varphi_{2} + \nabla \times \nabla \times (\eta_{2} \hat{z}), \\ \vec{u}_{3} & = \nabla \varphi_{3} + \nabla \times \nabla \times (\eta_{3} \hat{z}), \\ \end{aligned}$$

where $\varphi_{m}$ and $\eta_{m}$ represent the displacement potentials of compressional waves (P1, P2, and P3) and shear waves (S1 and S2), respectively. The subscript *m* = 1, 2, 3 refers to the solid grain frame, pore fluid, and hydrate, respectively. The displacement potential of compressional waves of each phase is composed of three parts, which are related to the P1 wave, P2 wave, and P3 wave. The displacement potentials of compressional waves in the frequency-wavenumber domain can be expressed as

3 $$\begin{aligned} \varphi_{1} (r,k_{z} ,\omega ) & = \frac{iF}{{2\rho_{f} \omega^{2} }}[B_{1} (k_{z} ,\omega )K_{0} (\alpha_{{{\text{P}}1}} r) + C_{1} (k_{z} ,\omega )K_{0} (\alpha_{{{\text{P}}2}} r) + D_{1} (k_{z} ,\omega )K_{0} (\alpha_{{{\text{P}}3}} r)], \\ \varphi_{2} (r,k_{z} ,\omega ) & = \frac{iF}{{2\rho_{f} \omega^{2} }}[B_{2} (k_{z} ,\omega )K_{0} (\alpha_{{{\text{P}}1}} r) + C_{2} (k_{z} ,\omega )K_{0} (\alpha_{{{\text{P}}2}} r) + D_{2} (k_{z} ,\omega )K_{0} (\alpha_{{{\text{P}}3}} r)], \\ \varphi_{3} (r,k_{z} ,\omega ) & = \frac{iF}{{2\rho_{f} \omega^{2} }}[B_{3} (k_{z} ,\omega )K_{0} (\alpha_{{{\text{P}}1}} r) + C_{3} (k_{z} ,\omega )K_{0} (\alpha_{{{\text{P}}2}} r) + D_{3} (k_{z} ,\omega )K_{0} (\alpha_{{{\text{P}}3}} r)], \\ \end{aligned}$$

where $B_{m}$, $C_{m}$ and $D_{m}$, $m = 1,\;2,\;3$ are the undetermined amplitudes of the three compressional waves, respectively; $\alpha_{{{\text{P}}1}}$, $\alpha_{{{\text{P}}2}}$ and $\alpha_{{{\text{P}}3}}$ represent the radial wave numbers of the three compressional waves. Moreover, in the frequency-wavenumber domain, the displacement potentials of shear waves are given as

4 $$\begin{aligned} \eta_{1} (r,\;k_{z} ,\;\omega ) & = \frac{iF}{{2\rho_{f} \omega^{2} }}[E_{1} (k_{z} ,\;\omega )K_{0} (\alpha_{{{\text{S}}1}} r) + F_{1} (k_{z} ,\;\omega )K_{0} (\alpha_{{{\text{S}}2}} r)], \\ \eta_{2} (r,\;k_{z} ,\;\omega ) & = \frac{iF}{{2\rho_{f} \omega^{2} }}[E_{2} (k_{z} ,\;\omega )K_{0} (\alpha_{{{\text{S}}1}} r) + F_{2} (k_{z} ,\;\omega )K_{0} (\alpha_{{{\text{S}}2}} r)], \\ \eta_{3} (r,\;k_{z} ,\;\omega ) & = \frac{iF}{{2\rho_{f} \omega^{2} }}[E_{3} (k_{z} ,\;\omega )K_{0} (\alpha_{{{\text{S}}1}} r) + F_{3} (k_{z} ,\;\omega )K_{0} (\alpha_{{{\text{S}}2}} r)], \\ \end{aligned}$$

where $E_{m}$ and $F_{m}$, $m = 1, \, 2, \, 3$ are the undetermined amplitudes of the two shear waves, respectively; $\alpha_{{{\text{S}}1}}$ and $\alpha_{{{\text{S2}}}}$ refer to the radial wave numbers of the two shear waves, respectively.

In the above formulas, $A_{f}$, $B_{m}$, $C_{m}$, $D_{m}$, $E_{m}$ and $F_{m}$ are the undetermined coefficients, which are determined by the boundary conditions of the borehole wall. Assuming that there is a relationship between the compressional wave displacement of each phase:

5 $$\varphi_{2} = l_{1} \varphi_{1} = l_{2} \varphi_{3} ,$$

then the following relationships can be obtained

6 $$\begin{aligned} B_{2} & = l_{11} B_{1} ,\;C_{2} = l_{12} C_{1} ,\;D_{2} = l_{13} D_{1} , \\ B_{3} & = \frac{{l_{11} }}{{l_{21} }}B_{1} ,\;C_{3} = \frac{{l_{12} }}{{l_{22} }}C_{1} ,\;D_{3} = \frac{{l_{13} }}{{l_{23} }}D_{1} . \\ \end{aligned}$$

Assuming that there is a relationship between the shear wave displacement of each phase:

7 $$\eta_{2} = l_{3} \eta_{1} = l_{4} \eta_{3} ,$$

then we can get

8 $$\begin{aligned} E_{2} & = l_{31} E_{1} ,\;F_{2} = l_{32} F_{1} , \\ E_{3} & = \frac{{l_{31} }}{{l_{41} }}E_{1} ,\;F_{3} = \frac{{l_{32} }}{{l_{42} }}F_{1} . \\ \end{aligned}$$

The solution process of the parameters $l_{ij} ,\;i = 1,\;2,\;3,\;4;\;j = 1,\;2,\;3$ is in Supplementary material.

The boundary conditions studied in this paper are the open hole boundary conditions, which can be written as

9 $$\begin{aligned} & u_{rin} = \phi_{s} u_{rsout} + \phi_{f} u_{rfout} + \phi_{h} u_{rhout} , \\ & - \phi_{s} P_{fin} = \sigma_{rrsout} , \\ & - \phi_{h} P_{fin} = \sigma_{rrhout} , \\ & P_{fin} = P_{fout} , \\ & \tau_{rzsout} = 0, \\ & \tau_{rzhout} = 0, \\ \end{aligned}$$

where the subscripts *in* and *out* refer to inside and outside the borehole wall, respectively; The subscripts *s*, *f* and *h* denote solid grains, pore fluid and hydrate, respectively. $\phi_{s}$, $\phi_{f}$ and $\phi_{h}$ refer to the volume fraction of solid grains, pore fluid and gas hydrate, respectively. $\phi_{s} + \phi_{f} + \phi_{h} = 1$, $\phi_{f} + \phi_{h} = \phi$. The first equation of Eq. (9) represents the continuity of fluid flow in the normal direction of the borehole wall. The second and the third equations show that the pressure of fluid in the well in the normal direction of the borehole wall is equal to the normal stress of the unit body in the gas hydrate formation outside the well in the same direction. The fourth equation represents that the pressure of the fluid in the well in the normal direction of the interface should be equal to that of the unit body in the porous formation outside the well. The fifth and sixth equations represent the continuity of shear stresses in the tangential direction of the borehole wall.

The number of boundary conditions is equal to the number of undetermined coefficients in the media inside and outside the well. The following linear equations can be obtained by combining the displacement of the compressional and shear wave potentials and the boundary conditions of each phase:

10 $$\left( {\begin{array}{*{20}c} {m_{11} } & {\quad m_{12} } & {\quad m_{13} } & {\quad m_{14} } & {\quad m_{15} } & {\quad m_{16} } \\ {m_{21} } & {\quad m_{22} } & {\quad m_{23} } & {\quad m_{24} } & {\quad m_{25} } & {\quad m_{26} } \\ {m_{31} } & {\quad m_{32} } & {\quad m_{33} } & {\quad m_{34} } & {\quad m_{35} } & {\quad m_{36} } \\ {m_{41} } & {\quad m_{42} } & {\quad m_{43} } & {\quad m_{44} } & {\quad m_{45} } & {\quad m_{46} } \\ {m_{51} } & {\quad m_{52} } & {\quad m_{53} } & {\quad m_{54} } & {\quad m_{55} } & {\quad m_{56} } \\ {m_{61} } & {\quad m_{62} } & {\quad m_{63} } & {\quad m_{64} } & {\quad m_{65} } & {\quad m_{66} } \\ \end{array} } \right)\left( {\begin{array}{*{20}c} A \\ {B_{m} } \\ {C_{m} } \\ {D_{m} } \\ {E_{m} } \\ {F_{m} } \\ \end{array} } \right) = \left( {\begin{array}{*{20}c} {b_{1} } \\ {b_{2} } \\ {b_{3} } \\ {b_{4} } \\ 0 \\ 0 \\ \end{array} } \right).$$

$m_{ij}$ and $b_{ij}$ are shown in Supplementary material. By equating the determinant of the $\left[ {m_{ij} } \right]_{6 \times 6}$ to $D\left( {k_{z} ,\omega } \right)$, and

$$N(k_{z} ,\;\omega ) = \left( {\begin{array}{*{20}c} {b_{1} } & {\quad m_{12} } & {\quad m_{13} } & {\quad m_{14} } & {\quad m_{15} } & {\quad m_{16} } \\ {b_{2} } & {\quad m_{22} } & {\quad m_{23} } & {\quad m_{24} } & {\quad m_{25} } & {\quad m_{26} } \\ {b_{31} } & {\quad m_{32} } & {\quad m_{33} } & {\quad m_{34} } & {\quad m_{35} } & {\quad m_{36} } \\ {b_{4} } & {\quad m_{42} } & {\quad m_{43} } & {\quad m_{44} } & {\quad m_{45} } & {\quad m_{46} } \\ 0 & {\quad m_{52} } & {\quad m_{53} } & {\quad m_{54} } & {\quad m_{55} } & {\quad m_{56} } \\ 0 & {\quad m_{62} } & {\quad m_{63} } & {\quad m_{64} } & {\quad m_{65} } & {\quad m_{66} } \\ \end{array} } \right),$$

the reflection coefficient $A_{f}$ in the borehole is given by

11 $$A_{f} (k_{z} ,\;\omega ) = \frac{{N(k_{z} ,\;\omega )}}{{D(k_{z} ,\;\omega )}}.$$

Substituting Eq. (11) into Eq. (1), the actual waveform received in the time domain can be obtained by the Fourier transform, as was done in an equivalent elastic formation with an embedded borehole^26^.

## Borehole waveforms in three-phase porous media

### Parameter selection

There are three friction coefficients in three-phase porous media: the friction coefficient between solid grain frame and pore fluid $b_{12}$^25^, the friction coefficient between hydrate and pore fluid $b_{23}$^25^, and the friction coefficient between solid grain frame and gas hydrate $b_{13}$^27^, which can be written as

12 $$b_{12} = \frac{{\eta_{f} \phi_{f}^{2} }}{{\kappa_{s} }},\;\;b_{23} = \frac{{\eta_{f} \phi_{f}^{2} }}{{\kappa_{h} }},\;\;b_{13} = 10^{11} \phi_{h}^{2} ,$$

where $\eta_{f}$ is the fluid viscosity (here is water), $\phi_{f}$ represents the porosity of the fluid, $\kappa_{s}$ and $\kappa_{h}$ refer to the relative permeabilities of solid grain frame and hydrate frame^25^, which can be expressed as

13 $$\kappa_{s} = \kappa_{s0} \left( {\frac{{\phi_{f} }}{\phi }} \right)^{3} ,\quad \kappa_{h} = \kappa_{h0} \left( {\frac{\phi }{{\phi_{h} }}} \right)^{2} \left( {\frac{{\phi_{f} }}{{\phi_{s} }}} \right)^{3} ,$$

where $\kappa_{s0}$ refers to the solid grain frame permeability, and $\kappa_{h0}$ is the gas hydrate frame permeability.

There are five kinds of body waves in a natural gas hydrate-bearing sediment^25^. The P1 and S1 waves are similar to the P wave and S wave in an elastic solid. The P2, S2 and P3 waves are three kinds of slow waves. The friction coefficient $b_{12}$ attenuates the P2 and S2 waves, which is related to the fluid viscosity, while $b_{13}$ makes the P3 wave attenuate^5^.

### Wave analysis

**Figure 2:**
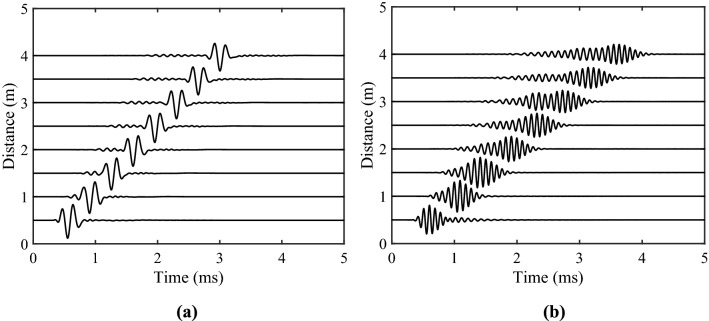
The borehole acoustic waveforms in the gas hydrate-bearing sediment. The center frequency of the source is (**a**) 6 kHz; (**b**) 10 kHz.

In this section, Borehole waveforms will be calculated with various acoustic parameters. The cosine envelope pulse is used as the sound source function when calculating the time domain waveform, which is given by

14 $$f(t) = \left\{ {\begin{array}{*{20}l} {\frac{1}{2}\left[ {1 + \cos \frac{2\pi }{{T_{c} }}\left( {t - \frac{{T_{c} }}{2}} \right)} \right]\cos 2\pi f_{0} \left( {t - \frac{{T_{c} }}{2}} \right)} & {\quad 0 \le t \le T_{c} } \\ 0 & {\quad t \le 0 \, \;or\; \, t \ge T_{c} } \\ \end{array} } \right.,$$

where $f_{0}$ and $T_{c}$ represent the center frequency and pulse width of the source, respectively. Figure 2a,b give the calculated waveforms of the RAI method at different locations from *z* = 0.5 m to *z* = 4 m in the gas hydrate-bearing sediment. The physical parameters of the sediment^4,25^ are given in Table 1. We calculated the RAI-modeled acoustic logs with different source domain frequencies of 6 kHz and 10 kHz, and they are full waveforms in the borehole. The pulse width *T*_*c*_ = 0.5 ms is employed in our calculation. As indicated in Fig. 2, there are three different wave groups of the waveforms: the first kind of compressional (P1) wave, the first kind of shear (S1) wave and the Stoneley wave. The amplitude of the P1 wave is small, while the S1 wave and Stoneley wave are apparently large. Moreover, it can be observed that with the increase of the source and receiver offsets, the attenuation of the Stoneley wave is more significant; The attenuation of the Stoneley wave at high frequency is greater than that at low frequency.

**Table 1:** Physical parameters of different phases.

Parameter	Value
Grain density (kg/m^3^)	2650
Water density (kg/m^3^)	1000
Gas hydrate density (kg/m^3^)	900
Grain bulk modulus (GPa)	38.7
Grain shear modulus (GPa)	39.6
Water bulk modulus (GPa)	2.25
Gas hydrate bulk modulus (GPa)	8.58
Gas hydrate shear modulus (GPa)	3.32

## Component wave calculations

### Path selection

Figure 2 shows the full waveforms received in the borehole, but the components of the full waveforms are superimposed on each other, which are difficult to identify. The Riemann surface is an effective tool for the analysis of multivalued functions^22,26^. For a defined $\omega$, the borehole characteristic function $D(k_{z} ,\;\omega )$ is a multivalued function of $k_{z}$ and it is appropriate to introduce Riemann surfaces for analysis. The analysis of Zhang et al.^22,23,26^ of borehole characteristic function and Riemann sheets for perfectly elastic borehole-formation configuration can be applied to the work of this paper. The potential function of the acoustic field of the borehole is a multivalued complex variable function, which is caused by the square root sign of the radial wave number $\alpha_{i} = \sqrt {k_{z}^{2} - k_{i}^{2} }$ and the second Bessel function $K_{0} (x)$ and $K_{1} (x)$. To calculate its poles, the multivalued function must be univalued. According to Cauchy theorem, the real axis integral is converted into the contribution of contour integral and pole residue. The path of integration coincides with the steepest descent path and passes through several different Riemann sheets^29^. A Riemann surface is composed of several Riemann sheets, and is single-valued on each Riemann sheet. Set $\theta_{j}$ as the argument of the radial wave number of five kinds of waves, $j = \alpha_{{{\text{P}}1}} ,\;\alpha_{{{\text{S}}1}} ,\;\alpha_{{{\text{P}}2}} ,\;\alpha_{{{\text{S}}2}} ,\;\alpha_{{{\text{P}}3}}$. Limit the range of argument $\theta_{j} \in (m\pi ,\;\pi + m\pi ),$ where *m* and *n* are integers, for any wavenumber *k* ($\omega$ is determined), the argument $\theta_{j}$ and the characteristic function $\varphi (\omega ,\;k_{z} )$ are uniquely determined. The domain of this single-valued function is called a sheet of the Riemann surface, different values of *m* and *n* correspond to different Riemann sheets. On each Riemann sheet, except for the poles and cut lines, the complex variable function is single value analytic. These five curves are also called SMF cut lines and their positions on different Riemann sheets are the same, $\alpha_{{{\text{P}}1}} ,\;\alpha_{{{\text{S}}1}} ,\;\alpha_{{{\text{P}}2}} ,\;\alpha_{{{\text{S}}2}} ,\;\alpha_{{{\text{P}}3}}$ are continuous, and each Riemann sheet is connected with each other along the cut line according to the principle of $\alpha_{{{\text{P}}1}} ,\;\alpha_{{{\text{S}}1}} ,\;\alpha_{{{\text{P}}2}} ,\;\alpha_{{{\text{S}}2}} ,\;\alpha_{{{\text{P}}3}}$ continuity to form a Riemann surface. Since the integers *m* and *n* can be arbitrarily taken, each Riemann sheet can be expressed as a Riemann sheet (*m*, *n*). Therefore, the entire Riemann surface consists of an infinite number of connected Riemann sheets. After obtaining the Riemann sheets and their connection rules, we can analyze the change of the Riemann sheets swept by the integration path after the integration path is converted from the real axis to the vertical cut line.

**Figure 3:**
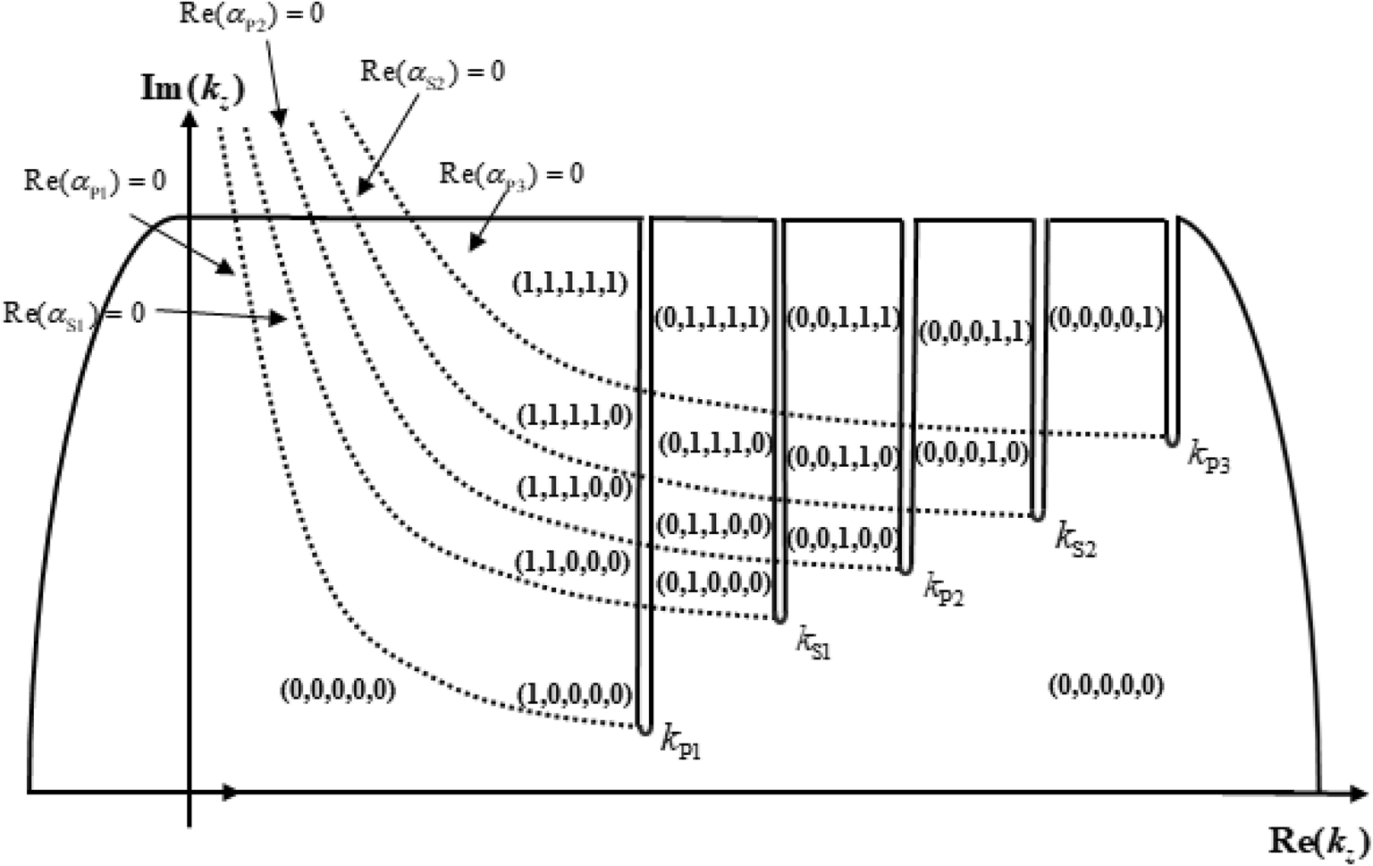
The Riemann sheets’ partition while integral contour traversing the SMF cut line.

As can be seen from Fig. 3, when the integration contour path crosses the SMF branch cut lines, there are 16 Riemann sheets for $D(k_{z} ,\;\omega )$ without repetition. The entire domain of the definition, that is, the entire Riemann surface of $D(k_{z} ,\;\omega )$ is composed of these 16 Riemann sheets bonded in turn. When SMF integration is adopted, the path of integration along the real axis is actually on (0, 0, 0, 0, 0) Riemann sheet. Since there are no complex poles on (0, 0, 0, 0, 0) Riemann sheet, the real axis integral of the acoustic field can be expressed as the path integral along the branch cut plus the contributions of the pole residues on the real axis, which does not involve the complex pole points on other Riemann sheets. Both the Stoneley and the pseudo-Rayleigh modes in the borehole are on the Riemann sheet of (0, 0, 0, 0, 0). The complex poles on other Riemannian sheets may correspond to the leaky modes in the borehole, which depends on the choice of the integral contour.

### Dispersion analysis

**Figure 4:**
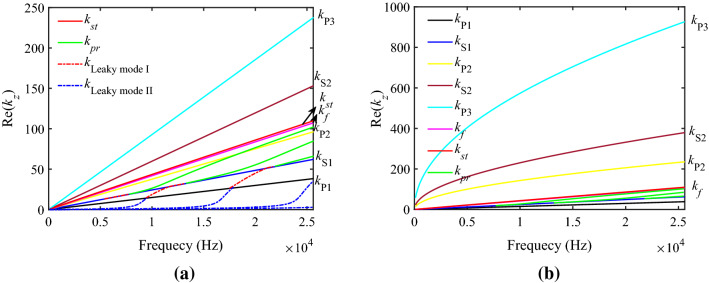
The dispersion curves with (**a**) the three friction coefficients being 0 and (**b**) the three friction coefficients being not 0.

In this paper, the solid grain frame permeability^25^ is chosen to be $\kappa_{s0} = 1.07 \times 10^{ - 13}$ m^2^. Figure 4 shows the dispersion curves of the different kinds of waves, which are obtained by solving dispersion equations. Three friction coefficients calculated in Fig. 4a are zero, while these friction coefficients in Fig. 4b are not zero. It can be seen from Fig. 4a,b that when the friction coefficients are zero, the slow waves are non-dispersive, the velocity of the P2 wave is less than that of the S1 wave and larger than that of the borehole fluid, and the velocity of the S2 wave is less than that of the borehole fluid and larger than that of the Stoneley wave. Meanwhile, the velocity of the P3 wave is less than that of the Stoneley wave. When the friction coefficients are not zero, the velocities of the three slow waves are less than the Stoneley wave velocity. Besides, the slow waves in the low-frequency range are dispersive while in the high-frequency range, they are not dispersive. The dispersions of the slow waves are caused by the friction coefficients between different phases. Moreover, the influence of friction coefficients on the dispersion of the slow waves in the low-frequency range is greater than that in the high frequency. The curvature degree of the real part of the poles with frequency on the dispersion curve reflects the strength of the dispersion of each mode. The Stoneley wave has no cut-off frequency and the dispersion curve is close to a straight line in our case. The pseudo-Rayleigh waves are more strongly dispersive and have multiple dispersion curves corresponding to multiple modes for different orders, each order of the pseudo-Rayleigh waves has a cut-off frequency at low frequencies and the real part of the wave number at the cut-off point tends to be close to that of the S1 wave. Furthermore, the Leaky mode I and the Leaky mode II can be observed in the dispersion curves. The Leaky mode I with cut-off frequency is between the S1 wave and P1 wave, each dispersion curve of the Leaky mode I has high and low cut-off frequencies, and the dispersion degree is larger than that of the pseudo-Rayleigh wave. The blue curves correspond to the multi-order Leaky mode II with a high cut-off frequency, which has a large dispersion.

### Excitation intensities and waveforms of mode waves

Numerical simulations and the component wave calculation of the full acoustic waveforms play an important role in understanding the propagation mechanisms of acoustic waves in the borehole and formation. In this section, the excitation intensity of each mode is calculated and discussed. Since the reflection coefficient in the borehole is given by Eq. (11), a vertical cut-line integral along the P1 wave and S1 wave branch points theoretically gives the corresponding excitation intensities for the P1 wave and S1 wave branch-cut, respectively. The branch-cut integral can be expressed as

15 $$I_{cut} (\omega ,\;z) = \int_{cut} {A_{f} } (k_{z} ,\;\omega )I_{0} (0)e^{{ik_{z} z}} dk_{z} .$$

The Romberg numerical integration is used along each perpendicular branch cut. When calculating this, it is important to note that when the cut line crosses the SMF cut line, the sign of the corresponding radial wavenumber should be changed. By integration, the excitation intensities of the body waves can be obtained.

**Figure 5:**
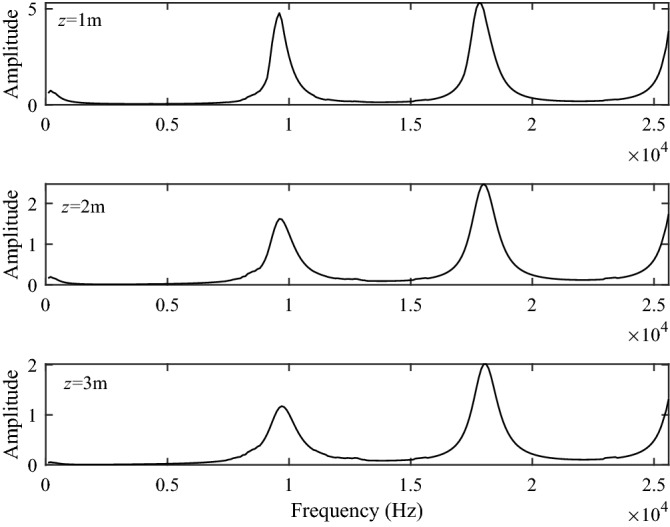
The excitation intensity of the P1 branch-cut integral.

**Figure 6:**
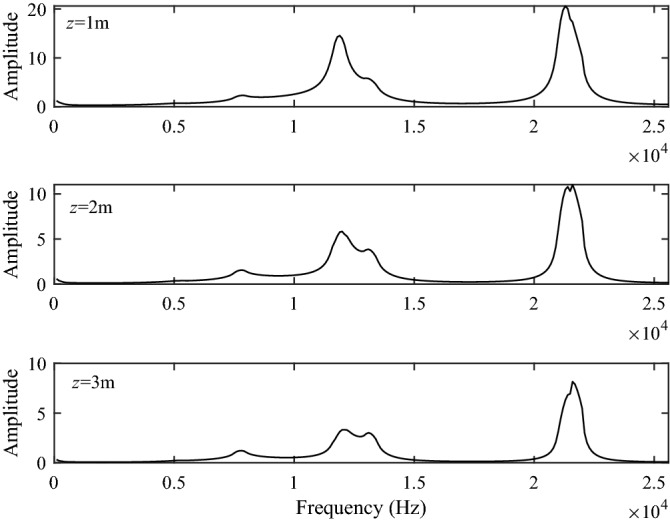
The excitation intensity of the S1 branch-cut integral.

Figures 5 and 6 show the excitation intensity curves of the P1 and S1 branch-cut integral at the source distances of *z* = 1 m, *z* = 2 m and *z* = 3 m, respectively. The excitation curves of the P1 and S1 branch-cut integral have a series of sharp strong excitation peaks at approximately the same interval, called resonance peaks, and the amplitude of the corresponding resonance peaks increases with the increasing frequency. at the same frequency, the excitation intensity decreases with increasing source distance. It is worth noting that, since the excitation intensity of slow waves is very small and the slow waves decay very quickly, the contribution to the total acoustic field of these slow waves propagating along the well axis can be neglected.

Once the frequency-excitation intensity relationship has been calculated for the wavelet, the time domain waveform of each wavelet in the borehole can be numerically simulated. When calculating the time domain waveform, we use the cosine envelope pulse as the source with Eq. (14). The frequency domain function of the corresponding source pulse $F(\omega )$ is obtained by inverting the Fourier transform of

16 $$F(\omega ) = \int_{ - \infty }^{\infty } f (t)e^{i\omega t} dt.$$

**Figure 7:**
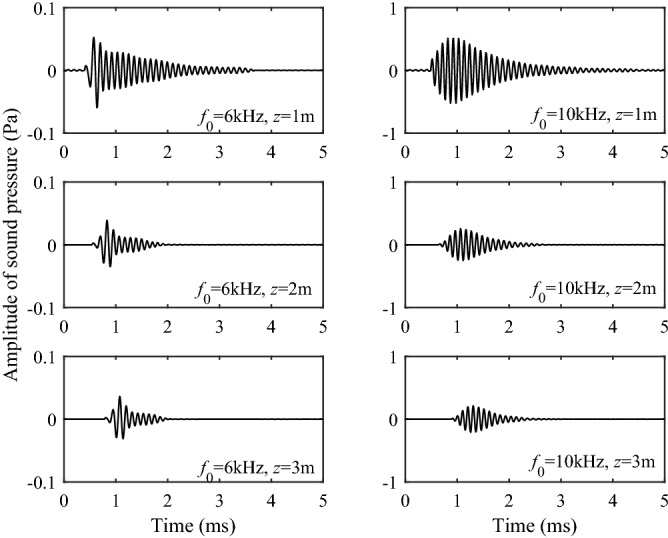
Waveforms in the time domain of the P1 branch-cut integral.

**Figure 8:**
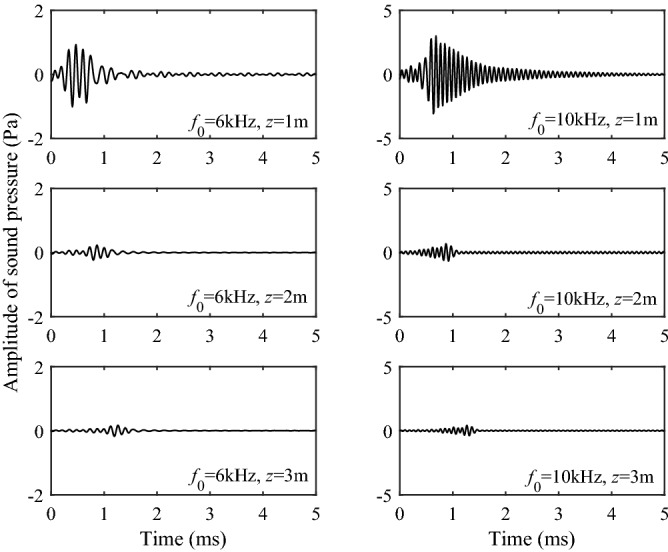
Waveforms in the time domain of the S1 branch-cut integral.

Based on the excitation intensity, we can obtain the time domain waveform of each wavelet by the Fourier transform for a defined point source pulse, which can be simulated numerically. This process can be expressed as

17 $$P(z,\;t) = \frac{1}{2\pi }\int_{ - \infty }^{\infty } I (\omega ,z) \cdot F(\omega )e^{ - i\omega t} d\omega ,$$

where $I(\omega )$ is the excitation intensity of each wavelet and $F(\omega )$ is the spectral expression of the source pulse. In the following Figs. 7 and 8, the waveforms of P1 and S1 branch-cut integral are compared at different source distances and different source center frequencies. As observed in Figs. 7 and 8, the waveform amplitudes of P1 branch-cut integral and S1 branch-cut integral decrease with the increase of the source distance and increase with the increase of the frequency. These phenomena are consistent with the previous analysis of the excitation intensity. It can be observed from Fig. 7 that when the source distance is large, the arrival of the waveforms of P1 branch-cut integral has a relatively obvious arrival point, but there are small interference signals in the waveform before the arrival point when the source distance is small. Figure 8 shows that the waveforms of S1 branch-cut integral obtained along the cut-line integration has meaningless non-physical signals, the interference signal before the arrival point is more obvious than that of the P1 branch-cut integral, so it cannot be directly separated from the full wave.

The excitation intensity of the Stoneley wave and the pseudo-Rayleigh wave can be given by the pole residue at the determined frequency, which can be expressed as

18 $$I_{pole \, } (k_{z} ,\;\omega ) = \left. {2\pi i \cdot e^{{ik_{z} z}} \frac{{N(k_{z} ,\;\omega )}}{{\partial D(k_{z} ,\;\omega )/\partial k_{z} }}} \right|_{{k_{z} = k_{n} }} .$$

Thus, for each determined source distance *z*, the excitation intensities curve for the Stoneley wave and the pseudo-Rayleigh wave are derived from the excitation intensity. The dimensional of the excitation intensity is length^2^ × time.

**Figure 9:**
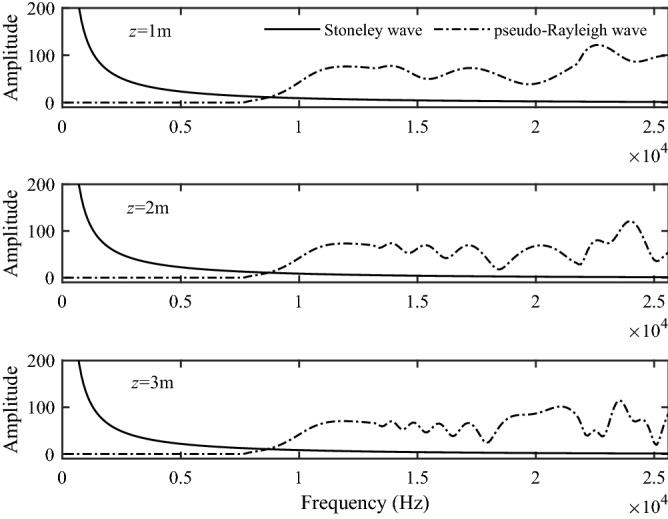
The excitation intensity of the Stoneley wave and the pseudo-Rayleigh wave.

Figure 9 shows the excitation intensity curves of the Stoneley and pseudo-Rayleigh waves at source distances of 1 m, 2 m and 3 m, respectively. As shown in Fig. 9, since there is no low-frequency cut-off frequency, the Stoneley wave is first excited at low frequency, and the excitation intensity is very large. Besides, the excitation intensity of the Stoneley wave decreases sharply with the increasing frequency. The pseudo-Rayleigh waves have a low-frequency cut-off frequency. The pseudo-Rayleigh waves in the frequency range smaller than the low-frequency cut-off frequency cannot be excited, and when the frequency is larger than the cut-off frequency, a zigzag curve is presented due to the iterative addition of the excitation intensity of each order of the pseudo-Rayleigh waves. As the source distance increases, the excitation intensity becomes weaker for both the Stoneley and pseudo-Rayleigh waves. Combined with Figs. 5 and 6, we can obtain that at the same source distance, the excitation intensities of the Stoneley wave and the pseudo-Rayleigh wave are larger than those of the P1 and S1 branch-cut integral.

**Figure 10:**
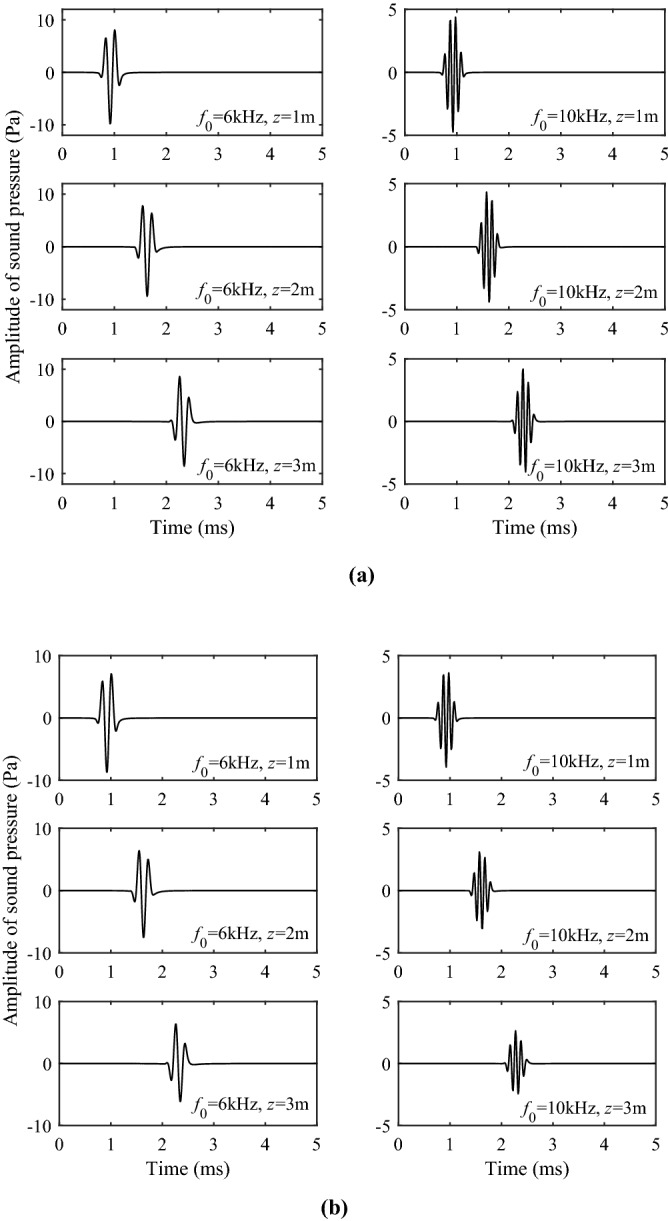
Waveforms in the time domain of the Stoneley wave. (**a**) the permeability of the solid grain is 10^−13^ m^2^; (**b**) the permeability of the solid grain is 10^−12^ m^2^.

**Figure 11:**
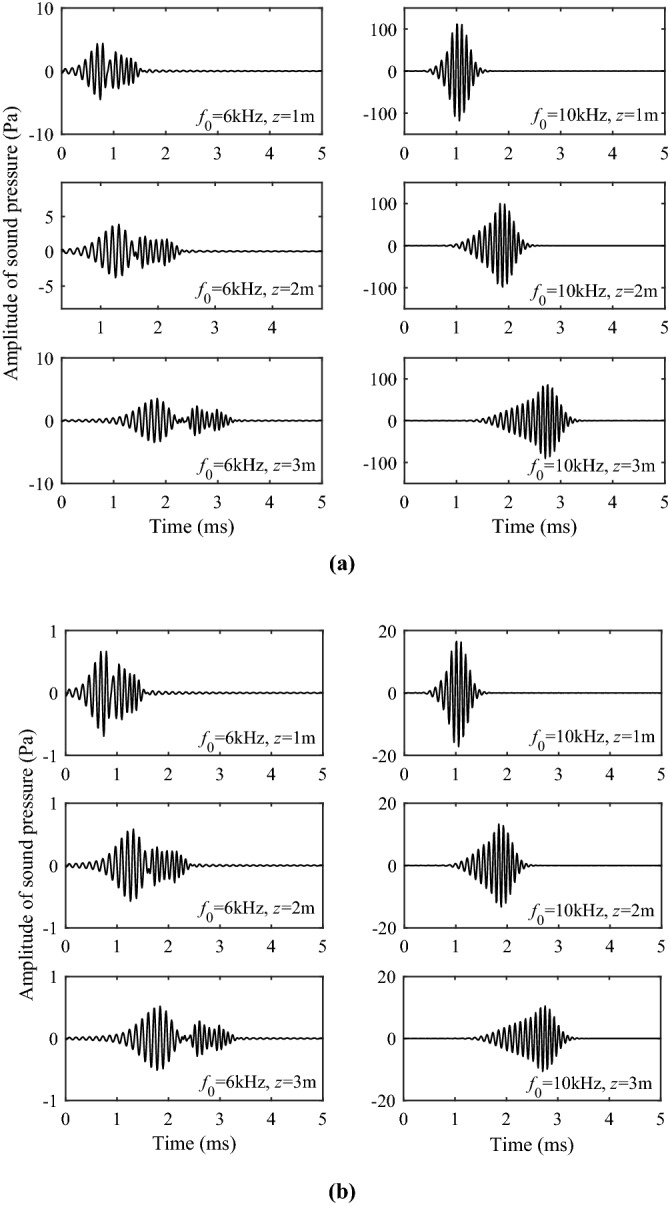
Waveforms in the time domain of the pseudo-Rayleigh wave. (**a**) the permeability of the solid grain is 10^−13^ m^2^; (**b**) the permeability of the solid grain is 10^−12^ m^2^.

Figure 10 shows the time-domain sound pressure for the Stoneley wave at different center frequencies and source distances. Figure 10a represents the solid grain permeability of 10^−13^ m^2^, while Fig. 10b represents the solid grain permeability of 10^−12^ m^2^. Figure 11 shows the time-domain sound pressure for the pseudo-Rayleigh wave at different center frequencies and source distances. It can be seen from Figs. 10 and 11 that the sound source with different central frequencies does not affect the travel time of the Stoneley and the pseudo-Rayleigh wave, but has a certain impact on the amplitude of the waveforms. This phenomenon can be explained by the excitation intensity curves in Fig. 9: When the center frequency of the source is 6 kHz, the excitation intensity of the Stoneley wave is large, and the Stoneley wave is easy to excite. As a result, the pressure amplitude of the Stoneley wave excited by the source at this center frequency is large. The pseudo-Rayleigh wave is less intense at around 6 kHz and has a lower amplitude. The same interpretation can explain the phenomenon that at a source center frequency of 10 kHz, the amplitude of the pseudo-Rayleigh wave is large while that of the Stoneley wave is small. This is due to the high excitation intensity of the pseudo-Rayleigh wave around 10 kHz and the low excitation intensity of the Stoneley wave around 10 kHz. In addition, as the pseudo-Rayleigh group consists of multiple modes, the velocity of each mode of the pseudo-Rayleigh waves is different due to the difference in the dispersion degree, which is manifested in the time domain waveform as the “spread” phenomenon. As the propagation distance increases, the “spread” phenomenon is more obvious. This phenomenon is not present in the Stoneley wave because the dispersion curve of the Stoneley wave has only a single branch. As the source distance increases, the amplitude of the Stoneley wave and the pseudo-Rayleigh wave decays, which corresponds to the previous analysis of the excitation intensity. The normal modes in a borehole of a three-phase porous formation are attenuated due to the relative movement of different phases as the wave propagates through the porous medium. In addition, The greater the solid grain permeability, the smaller the Stoneley wave amplitude, and the more obvious the attenuation phenomenon with the increase of the source distance.

**Figure 12:**
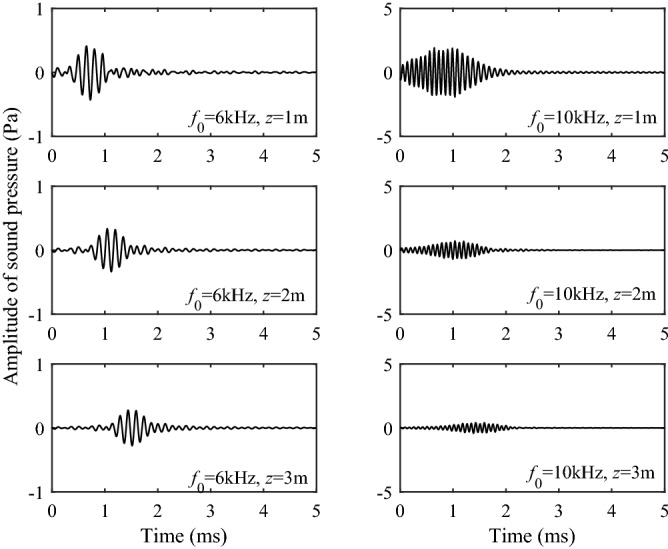
Waveforms in the time domain of the Leaky mode I.

According to the work of Zhang et al.^22,23,26,28^, He^29^ and Zheng^24^, when the center frequency of the source is high, the results of branch-cut integration of S1 wave cannot be used to explain the wave. The reason is that the branch-cut integral ignores the contribution of the pole near the branch cut, and the calculation of the integral alone cannot truly reflect the waveforms of the S1 wave in the propagation. Therefore, the contribution of the pole near the branch-cut line should be considered when the real axis integral is converted into the contour integral as shown in Fig. 3. According to the residue theorem, the complex poles on the other Riemann sheets corresponds to the geometric attenuation wave and the Leaky mode of the borehole acoustic field. The contribution of leaky mode I on the (0, 1, 0, 0, 0) sheet and the pseudo-Rayleigh wave on the (0, 0, 0, 0, 0) sheet adjacent to the branch of the S1 wave must be considered when calculating the time-domain waveform of the S1 wave. Figure 12 shows the waveforms of the Leaky mode I under different frequencies and source distances. It can be seen from the figure that the Leaky mode I has a large amplitude, which cannot be ignored.

**Figure 13:**
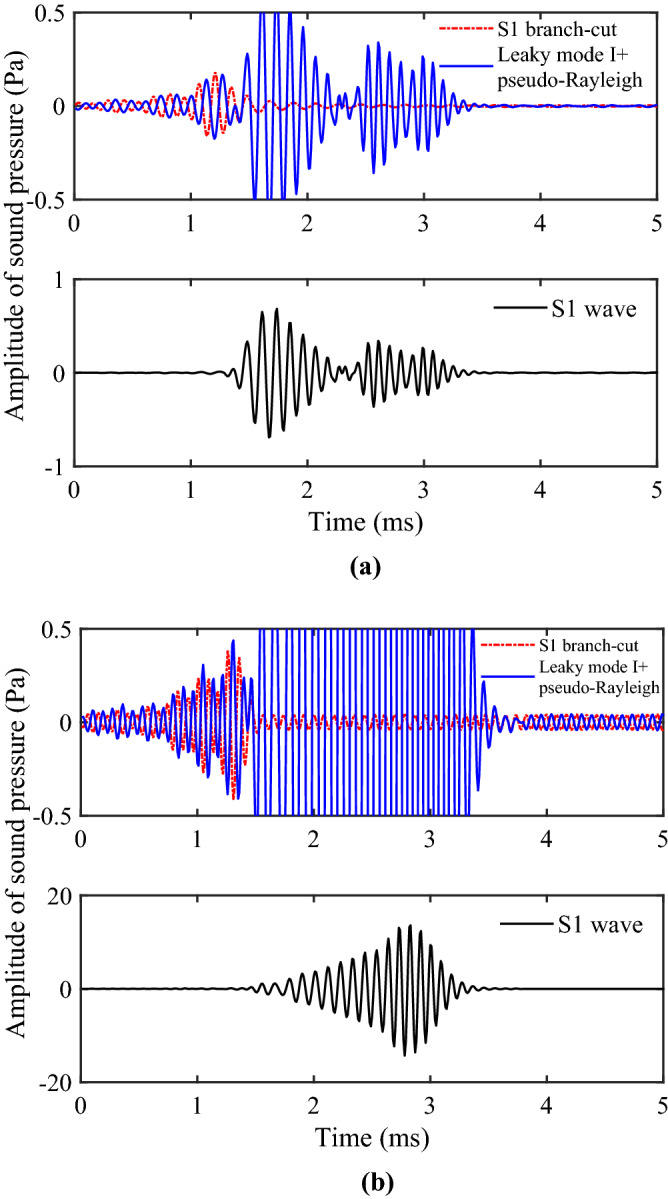
The S1 wave and its contributing components. The S1 wave is obtained by summing up the component parts of S1 branch-cut, the Leaky mode I and the pseudo-Rayleigh wave. (**a**) 6 kHz; (**b**) 10 kHz.

Figure 13 shows the S1 wave group and its contributing components at 6 kHz and 10 kHz. The S1 wave group is obtained by summing up the component parts of S1 branch-cut, the Leaky mode I, and the pseudo-Rayleigh wave. The Leaky mode I is the contribution of the complex pole on the (0, 1, 0, 0, 0) sheet, and the pseudo-Rayleigh wave represents the contribution of the real pole on the (0, 0, 0, 0, 0) sheet. The results indicate that combined with the contribution of poles, the S1 wave group can be effectively obtained, eliminating the interference of non-physical signals.

**Figure 14:**
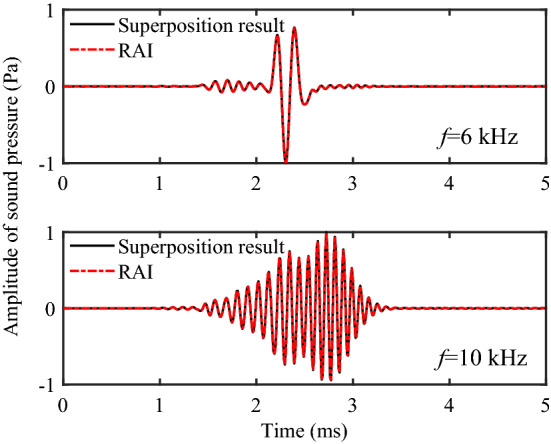
Comparison of the superposition of each component and the real axis integration.

Figure 14 shows the comparison between the superposition results of each component and the real axis integration results at 6 kHz and 10 kHz. It can be observed that the two results are in good agreement, which proves the correctness of pole and branch-cut integral calculation and provides a theoretical basis for the evaluation of reservoir parameters by using the component information.

## Conclusion

Based on the Carcione–Leclaire three-phase theory, we described the displacement potentials of each phase (solid grain frame, gas hydrate, and pore fluid) of a Biot-type three-phase porous medium. Besides, we constructed the boundary conditions of fluid and three-phase porous media. Combining the above two aspects, we proposed a RAI method to analytically solve the acoustic field in a borehole with a gas hydrate-bearing sediment.

According to the analysis of the Riemann surface of the borehole characteristic function surrounded by porous media containing two solids and one fluid, the contribution of the branch points and poles is analyzed when the real axis integral converted into the branch-cut integral. For the first time, the P1 branch-cut, S1 branch-cut, Stoneley wave, pseudo-Rayleigh wave and Leaky mode I are calculated separately, with their dispersion, excitation intensity, and component wave being analyzed. The superposition of the S1 branch-cut, the pseudo-Rayleigh wave and Leaky mode I can effectively eliminate interference signals and obtain the clean S1 wave group. To illustrate our results, the full waveform summed up from the component waves is compared with that simulated by the RAI method. Two full waveforms agree well, which proves the effectiveness of the component wave analysis. Once the component waves are effectively separated from the full wave, the variation of the component waves with the formation parameters outside the borehole can be analyzed to better obtain reservoir information from acoustic logging data, which provides a theoretical guide for hydrate exploration and extraction.

## Supplementary Information

Supplementary Information.

## Data Availability

The datasets used and/or analyzed during the current study available from the corresponding author on reasonable.
