# Supplementary material for: Component wave calculation and analysis of the acoustic field in a borehole within a three-phase porous medium
Source: Sci Rep. 2023 May 11;13:7646. doi: 10.1038/s41598-023-33709-8 (PMC10175551; doi:10.1038/s41598-023-33709-8)
Supplement: Supplementary file 1 — Supplementary Information. [file 41598_2023_33709_MOESM1_ESM.docx]

**Supplementary Material**

Eqs (5) and (6) show the relationship between the unknown coefficients in displacements of compressional and shear waves of each phase. Therefore, in the frequency-wavenumber domain, the displacement potentials of compressional and shear waves can be expressed as

(A1)

$\begin{matrix} \eta_{1}(r,k,\omega)\text{=}\frac{iF}{2\rho_{f}\omega^{2}}[E_{1}K_{0}(\alpha_{\text{S1}}r)+F_{1}K_{0}(\alpha_{\text{S2}}r)], \\ \eta_{2}(r,k,\omega)\text{=}\frac{iF}{2\rho_{f}\omega^{2}}[l_{31}E_{1}K_{0}(\alpha_{\text{S1}}r)+l_{32}F_{1}K_{0}(\alpha_{\text{S2}}r)], \\ \eta_{3}(r,k,\omega)\text{=}\frac{iF}{2\rho_{f}\omega^{2}}[\frac{l_{31}}{l_{41}}E_{1}K_{0}(\alpha_{\text{S1}}r)+\frac{l_{32}}{l_{42}}F_{1}K_{0}(\alpha_{\text{S2}}r)]. \end{matrix}$ (A2)

Substitute the displacement potentials of compressional and shear waves into the boundary condition respectively. For the first boundary condition at the borehole wall,

(A3)

where

(A4)

Thus, substitute Eqs (1) and (A4) into Eq. (A3), we can obtain that

(A5)

For the second boundary condition,

(A6)

The fluid pressure in the borehole $P_{f}$ is given by

(A7)

The radial stress of solid grain frame in natural gas hydrate formation $\sigma_{rrsout}$ can be expressed as

$\begin{matrix} \sigma_{rrsout}=K_{1}(e_{rr}^{(1)}+e_{\theta\theta}^{(1)}+e_{zz}^{(1)})+C_{12}(e_{rr}^{(2)}+e_{\theta\theta}^{(2)}+e_{zz}^{(2)})+C_{13}(e_{rr}^{(3)}+e_{\theta\theta}^{(3)}+e_{zz}^{(3)}) \\ \text{ }+2\mu_{1}[e_{rr}^{(1)}-\frac{1}{3}(e_{rr}^{(1)}+e_{\theta\theta}^{(1)}+e_{zz}^{(1)})]+\mu_{13}[e_{rr}^{(3)}-\frac{1}{3}(e_{rr}^{(3)}+e_{\theta\theta}^{(3)}+e_{zz}^{(3)})] \\ \text{ }=(K_{1}+\frac{4}{3}\mu_{1})\nabla\cdot\vec{u}_{1}+C_{12}\nabla\cdot\vec{u}_{2}+(C_{13}+\frac{2}{3}\mu_{13})\nabla\cdot\vec{u}_{3}\text{ } \\ \text{ -2}\mu_{1}\text{(}e_{\theta\theta}^{(1)}+e_{zz}^{(1)})\text{ -}\mu_{13}\text{(}e_{\theta\theta}^{(3)}+e_{zz}^{(3)}),\text{ } \end{matrix}$ (A8)

where $K_{1}$ denotes the effective bulk modulus of solid grain frame; $C_{12}$ and $C_{13}$ refer to the elastic coupling between solid grain frame and pore fluid, the solid grain frame and gas hydrate, respectively; $\mu_{1}$ is the effective shear modulus of solid grain frame; $\mu_{13}$ represents the shear coupling between solid grain frame and gas hydrate; $e_{rr}$, $e_{\theta\theta}$ and $e_{zz}$ are the strain tensors in three directions, which can be expressed as

$e_{rr}=\frac{\partial u_{r}}{\partial r},\text{ }e_{\theta\theta}=\frac{u_{r}}{r},\text{ }e_{zz}=\frac{\partial u_{z}}{\partial r},$ (A9)

where $u_{z}$ is the displacement in the *z*-direction, which is defined as

(A10)

Besides,

(A11)

and

$\begin{matrix} k_{\text{P}}^{2}=k_{z}^{2}-\alpha^{2}, \\ k_{\text{S}}^{2}=k_{z}^{2}-\beta^{2}. \end{matrix}$ (A12)

By combining Eq. (A8) with (A9)–(A12), we can deduce

$\begin{matrix} \sigma_{rrsout}=\frac{iF}{2\rho_{f}\omega^{2}}\text{\{}-[K_{1}+\frac{4}{3}\mu_{1}+l_{11}C_{12}+\frac{l_{11}}{l_{21}}(C_{13}+\frac{2}{3}\mu_{13})]k_{\text{P}1}^{2}K_{0}(\alpha_{1}r)B_{1} \\ -[K_{1}+\frac{4}{3}\mu_{1}+l_{12}C_{12}+\frac{l_{12}}{l_{22}}(C_{13}+\frac{2}{3}\mu_{13})]k_{\text{P2}}^{2}K_{0}(\alpha_{2}r)C_{1} \\ -[K_{1}+\frac{4}{3}\mu_{1}+l_{13}C_{12}+\frac{l_{13}}{l_{23}}(C_{13}+\frac{2}{3}\mu_{13})]k_{\text{P3}}^{2}K_{0}(\alpha_{3}r)D_{1} \\ +(2\mu_{1}+\frac{l_{11}}{l_{21}}\mu_{13})k_{z}^{2}K_{0}(\alpha_{1}r)B_{1}+(2\mu_{1}+\frac{l_{12}}{l_{22}}\mu_{13})k_{z}^{2}K_{0}(\alpha_{2}r)C_{1} \\ +(2\mu_{1}+\frac{l_{13}}{l_{23}}\mu_{13})k_{z}^{2}K_{0}(\alpha_{2}r)D_{1}+(2\mu_{1}+\frac{l_{31}}{l_{41}}\mu_{13})ik_{z}\beta_{1}^{2}K_{0}(\beta_{1}r)E_{1} \\ +(2\mu_{1}+\frac{l_{32}}{l_{42}}\mu_{13})ik_{z}\beta_{2}^{2}K_{0}(\beta_{2}r)F_{1}+\frac{1}{r}(2\mu_{1}+\frac{l_{11}}{l_{21}}\mu_{13})\alpha_{1}K_{1}(\alpha_{1}r)B_{1} \\ +\frac{1}{r}(2\mu_{1}+\frac{l_{12}}{l_{22}}\mu_{13})\alpha_{2}K_{1}(\alpha_{2}r)C_{1}+\frac{1}{r}(2\mu_{1}+\frac{l_{12}}{l_{22}}\mu_{13})\alpha_{2}K_{1}(\alpha_{2}r)C_{1} \\ +\frac{1}{r}(2\mu_{1}+\frac{l_{13}}{l_{23}}\mu_{13})\alpha_{3}K_{1}(\alpha_{3}r)D_{1}+\frac{1}{r}(2\mu_{1}+\frac{l_{31}}{l_{41}}\mu_{13})ik_{z}\beta_{1}K_{1}(\beta_{1}r)E_{1} \\ +\frac{1}{r}(2\mu_{1}+\frac{l_{32}}{l_{42}}\mu_{13})ik_{z}\beta_{2}K_{1}(\beta_{2}r)F_{1}. \end{matrix}$ (A13)

According to the open hole boundary condition, combined with the acoustic field in the borehole, the boundary condition of the solid grain frame at the borehole wall can be expressed as

(A14)

For the boundary condition of the gas hydrate

(A15)

the solution process is the same as that of the solid frame. The radial stress of gas hydrate is given by

$\begin{matrix} \sigma_{rrhout}=K_{3}(e_{rr}^{(3)}+e_{\theta\theta}^{(3)}+e_{zz}^{(3)})+C_{13}(e_{rr}^{(1)}+e_{\theta\theta}^{(1)}+e_{zz}^{(1)})+C_{23}(e_{rr}^{(2)}+e_{\theta\theta}^{(2)}+e_{zz}^{(2)}) \\ \text{ }+2\mu_{3}[e_{rr}^{(3)}-\frac{1}{3}(e_{rr}^{(3)}+e_{\theta\theta}^{(3)}+e_{zz}^{(3)})]+\mu_{13}[e_{rr}^{(1)}-\frac{1}{3}(e_{rr}^{(1)}+e_{\theta\theta}^{(1)}+e_{zz}^{(1)})] \\ \text{ }=(K_{3}+\frac{4}{3}\mu_{3})\nabla\cdot\vec{u}_{3}+C_{23}\nabla\cdot\vec{u}_{2}+(C_{13}+\frac{2}{3}\mu_{13})\nabla\cdot\vec{u}_{3} \\ \text{ }-2\mu_{3}\text{(}e_{\theta\theta}^{(3)}+e_{zz}^{(3)})\text{ -}\mu_{13}\text{(}e_{\theta\theta}^{(1)}+e_{zz}^{(1)}), \end{matrix}$ (A16)

where $K_{3}$ refers to the effective bulk modulus of the hydrate frame; $\mu_{3}$ is the effective shear modulus of the hydrate frame; $C_{23}$ denotes the elastic coupling between pore fluid and gas hydrate. The boundary condition of the gas hydrate phase at the borehole wall can be expressed as

(A17)

For the boundary condition of the pore fluid

$P_{fin}=P_{fout},$ (A18)

the fluid pressure of the gas hydrate reservoir outside the borehole is

(A19)

where $K_{2}$ denotes the effective bulk modulus of the pore fluid. Thus,

(A20)

For the boundary condition

$\sigma_{rz}^{(1)}=0,$ (A21)

$\sigma_{rz}^{(3)}=0.$ (A22)

The shear stresses of the solid grain frame and hydrate frame outside the well can be expressed as

$\sigma_{rz}^{(1)}=\mu_{1}(\frac{\partial u_{r}^{(1)}}{\partial z}+\frac{\partial u_{z}^{(1)}}{\partial r})+\frac{1}{2}\mu_{13}(\frac{\partial u_{r}^{(3)}}{\partial z}+\frac{\partial u_{z}^{(3)}}{\partial r}),$ (A23)

$\sigma_{rz}^{(3)}=\mu_{3}(\frac{\partial u_{r}^{(3)}}{\partial z}+\frac{\partial u_{z}^{(3)}}{\partial r})+\frac{1}{2}\mu_{13}(\frac{\partial u_{r}^{(1)}}{\partial z}+\frac{\partial u_{z}^{(1)}}{\partial r}).$ (A24)

Besides,

(A25)

the boundary condition of the shear stress of the solid grain frame at the borehole wall can be expressed as

$\begin{matrix} -(\mu_{1}+\frac{1}{2}\frac{l_{11}}{l_{21}}\mu_{13})ik_{z}\alpha_{1}K_{1}(\alpha_{1}a)B_{1}-(\mu_{1}+\frac{1}{2}\frac{l_{12}}{l_{22}}\mu_{13})ik_{z}\alpha_{2}K_{1}(\alpha_{2}a)C_{1} \\ -(\mu_{1}+\frac{1}{2}\frac{l_{13}}{l_{23}}\mu_{13})ik_{z}\alpha_{3}K_{1}(\alpha_{3}a)D_{1}+(\mu_{1}+\frac{1}{2}\frac{l_{31}}{l_{41}}\mu_{13})k_{z}^{2}\beta_{1}K_{1}(\beta_{1}a)E_{1} \\ +(\mu_{1}+\frac{1}{2}\frac{l_{32}}{l_{42}}\mu_{13})k_{z}^{2}\beta_{2}K_{1}(\beta_{2}a)F_{1}-(\mu_{1}+\frac{1}{2}\frac{l_{11}}{l_{21}}\mu_{13})ik_{z}\alpha_{1}K_{1}(\alpha_{1}a)B_{1} \\ -(\mu_{1}+\frac{1}{2}\frac{l_{12}}{l_{22}}\mu_{13})ik_{z}\alpha_{2}K_{1}(\alpha_{2}a)C_{1}-(\mu_{1}+\frac{1}{2}\frac{l_{13}}{l_{23}}\mu_{13})ik_{z}\alpha_{3}K_{1}(\alpha_{3}a)D_{1} \\ +(\mu_{1}+\frac{1}{2}\frac{l_{31}}{l_{41}}\mu_{13})\beta_{1}^{2}\beta_{1}K_{1}(\beta_{1}a)E_{1}+(\mu_{1}+\frac{1}{2}\frac{l_{32}}{l_{42}}\mu_{13})\beta_{2}^{2}\beta_{2}K_{1}(\beta_{2}a)F_{1}=0. \end{matrix}$ (A26)

Similarly, the boundary condition of the shear stress of the gas hydrate at the borehole wall can be expressed as

$\begin{matrix} -(\mu_{3}+\frac{1}{2}\frac{l_{11}}{l_{21}}\mu_{13})ik_{z}\alpha_{1}K_{1}(\alpha_{1}a)B_{1}-(\mu_{3}+\frac{1}{2}\frac{l_{12}}{l_{22}}\mu_{13})ik_{z}\alpha_{2}K_{1}(\alpha_{2}a)C_{1} \\ -(\mu_{3}+\frac{1}{2}\frac{l_{13}}{l_{23}}\mu_{13})ik_{z}\alpha_{3}K_{1}(\alpha_{3}a)D_{1}+(\mu_{3}+\frac{1}{2}\frac{l_{31}}{l_{41}}\mu_{13})k_{z}^{2}\beta_{1}K_{1}(\beta_{1}a)E_{1} \\ +(\mu_{3}+\frac{1}{2}\frac{l_{32}}{l_{42}}\mu_{13})k_{z}^{2}\beta_{2}K_{1}(\beta_{2}a)F_{1}-(\mu_{3}+\frac{1}{2}\frac{l_{11}}{l_{21}}\mu_{13})ik_{z}\alpha_{1}K_{1}(\alpha_{1}a)B_{1} \\ -(\mu_{3}+\frac{1}{2}\frac{l_{12}}{l_{22}}\mu_{13})ik_{z}\alpha_{2}K_{1}(\alpha_{2}a)C_{1}-(\mu_{3}+\frac{1}{2}\frac{l_{13}}{l_{23}}\mu_{13})ik_{z}\alpha_{3}K_{1}(\alpha_{3}a)D_{1} \\ +(\mu_{3}+\frac{1}{2}\frac{l_{31}}{l_{41}}\mu_{13})\beta_{1}^{2}\beta_{1}K_{1}(\beta_{1}a)E_{1}+(\mu_{3}+\frac{1}{2}\frac{l_{32}}{l_{42}}\mu_{13})\beta_{2}^{2}\beta_{2}K_{1}(\beta_{2}a)F_{1}=0. \end{matrix}$ (A27)

Finally, we can get the values of each parameter in the *M* matrix, which are given by

(A28)

(A29)

(A30)

(A31)

$\begin{matrix} m_{51}=0, \\ m_{52}=-2ik_{z}\alpha_{\text{P1}}(\mu_{1}+\frac{1}{2}\frac{l_{11}}{l_{21}}\mu_{13})K_{1}(\alpha_{\text{P1}}a), \\ m_{53}=-2ik_{z}\alpha_{\text{P2}}(\mu_{1}+\frac{1}{2}\frac{l_{12}}{l_{22}}\mu_{13})K_{1}(\alpha_{\text{P2}}a), \\ m_{54}=-2ik_{z}\alpha_{\mathrm{P}3}(\mu_{1}+\frac{1}{2}\frac{l_{13}}{l_{23}}\mu_{13})K_{1}(\alpha_{\text{P3}}a), \\ m_{55}=\alpha_{\text{S1}}(\mu_{1}+\frac{1}{2}\frac{l_{31}}{l_{41}}\mu_{13})(k_{z}^{2}+\alpha_{\text{S1}}^{2})K_{1}(\alpha_{\text{S1}}a), \\ m_{56}=\alpha_{\text{S2}}(\mu_{1}+\frac{1}{2}\frac{l_{32}}{l_{42}}\mu_{13})(k_{z}^{2}+\alpha_{\text{S2}}^{2})K_{1}(\alpha_{\text{S2}}a), \end{matrix}$ (A32)

$\begin{matrix} m_{61}=0, \\ m_{62}=-2ik_{z}\alpha_{\text{P1}}(\mu_{13}+\frac{1}{2}\frac{l_{11}}{l_{21}}\mu_{3})K_{1}(\alpha_{\text{P1}}a), \\ m_{63}=-2ik_{z}\alpha_{\text{P2}}(\mu_{13}+\frac{1}{2}\frac{l_{12}}{l_{22}}\mu_{3})K_{1}(\alpha_{\text{P2}}a), \\ m_{64}=-2ik_{z}\alpha_{\text{P3}}(\mu_{13}+\frac{1}{2}\frac{l_{13}}{l_{23}}\mu_{3})K_{1}(\alpha_{\text{P3}}a), \\ m_{65}=\alpha_{\text{S1}}(\mu_{13}+\frac{1}{2}\frac{l_{31}}{l_{41}}\mu_{3})(k_{z}^{2}+\alpha_{\text{S1}}^{2})K_{1}(\alpha_{\text{S1}}a), \\ m_{66}=\alpha_{\text{S2}}(\mu_{13}+\frac{1}{2}\frac{l_{32}}{l_{42}}\mu_{3})(k_{z}^{2}+\alpha_{\text{S2}}^{2})K_{1}(\alpha_{\text{S2}}a). \end{matrix}$ (A33)

In addition,

(A34)

Among the unknowns of the above parameters, $l_{11}$, $l_{12}$, $l_{13}$, and $l_{21}$, $l_{22}$, $l_{23}$ correspond to three kinds of compressional waves, respectively; $l_{31}$, $l_{32}$ and $l_{41}$, $l_{42}$ correspond to two kinds of shear waves, respectively. They are constants determined by the medium parameters.

The total stress component of the natural gas hydrate reservoir is given by

(A35)

The motion equation can be expressed as

(A36)

where $\rho_{ij}$ refers to the coupling mass density. By substituting the equation of motion into the constitutive relation, we can obtain

(A37)

Similarly, the constitutive relations of pore fluid and hydrate phase are defined as

$P_{f}=C_{12}\nabla\cdot\vec{u}_{1}+K_{2}\nabla\cdot\vec{u}_{2}+C_{23}\nabla\cdot\vec{u}_{3},$ (A38)

$\begin{matrix} \sigma_{ij}^{(3)}=(K_{3}\nabla\cdot\vec{u}_{3}+C_{13}\nabla\cdot\vec{u}_{1}+C_{23}\nabla\cdot\vec{u}_{2})\delta_{ij} \\ \text{ +2}\mu_{3}(e_{ij}^{(3)}-\frac{1}{3}\delta_{ij}\nabla\cdot\vec{u}_{3})+\mu_{13}(e_{ij}^{(1)}-\frac{1}{3}\delta_{ij}\nabla\cdot\vec{u}_{1}) \\ \text{ =[}(K_{3}-\frac{2}{3}\mu_{3})\nabla\cdot\vec{u}_{3}+C_{23}\nabla\cdot\vec{u}_{2} \\ \text{ }+(C_{13}-\frac{1}{3}\mu_{13})\nabla\cdot\vec{u}_{1}]\delta_{ij}\text{+2}\mu_{3}e_{ij}^{(3)}+\mu_{13}e_{ij}^{(1)}. \end{matrix}$ (A39)

The motion equations of pore fluid and hydrate frame are

(A40)

(A41)

By substituting the equations of motion into the constitutive relations, we can obtain

(A42)

(A43)

Eqs (A37), (A42) and (A43) constitute the wave differential equation, the vector form in the frequency domain is given by

$\begin{matrix} (\mu_{1}+\frac{1}{2}\mu_{13})\nabla^{2}\vec{u}_{1}+(\mu_{3}+\frac{1}{2}\mu_{13})\nabla^{2}\vec{u}_{3}+(K_{1}+C_{12}+C_{13}+\frac{1}{3}\mu_{1}+\frac{1}{6}\mu_{13})\nabla(\nabla\cdot\vec{u}_{1}) \\ +(K_{2}+C_{12}+C_{23})\nabla(\nabla\cdot\vec{u}_{2})+(K_{3}+C_{13}+C_{23}+\frac{1}{3}\mu_{3}+\frac{1}{6}\mu_{13})\nabla(\nabla\cdot\vec{u}_{3}) \\ +(\rho_{11}+\rho_{12}+\rho_{13})\omega^{2}\vec{u}_{1}+(\rho_{12}+\rho_{22}+\rho_{23})\omega^{2}\vec{u}_{2}+(\rho_{13}+\rho_{23}+\rho_{33})\omega^{2}\vec{u}_{3}=0, \end{matrix}$(A44)

$\begin{matrix} \mu_{3}\nabla^{2}\vec{u}_{3}+\frac{1}{2}\mu_{13}\nabla^{2}\vec{u}_{1}+(K_{3}\text{+}\frac{1}{3}\mu_{3})\nabla(\nabla\cdot\vec{u}_{3}) \\ +C_{23}\nabla(\nabla\cdot\vec{u}_{3})+[C_{13}+\frac{1}{6}\mu_{13}]\nabla(\nabla\cdot\vec{u}_{1}) \\ +\rho_{13}\omega^{2}\vec{u}_{1}+\rho_{23}\omega^{2}\vec{u}_{2}+\rho_{33}\omega^{2}\vec{u}_{3} \\ -i\omega b_{23}(\vec{u}_{2}-\vec{u}_{3})+i\omega b_{13}(\vec{u}_{3}-\vec{u}_{1}). \end{matrix}$ (A45)

$\begin{matrix} \mu_{3}\nabla^{2}\vec{u}_{3}+\frac{1}{2}\mu_{13}\nabla^{2}\vec{u}_{1}+(K_{3}\text{+}\frac{1}{3}\mu_{3})\nabla(\nabla\cdot\vec{u}_{3}) \\ +C_{23}\nabla(\nabla\cdot\vec{u}_{3})+[C_{13}+\frac{1}{6}\mu_{13}]\nabla(\nabla\cdot\vec{u}_{1}) \\ +\rho_{13}\omega^{2}\vec{u}_{1}+\rho_{23}\omega^{2}\vec{u}_{2}+\rho_{33}\omega^{2}\vec{u}_{3} \\ -i\omega b_{23}(\vec{u}_{2}-\vec{u}_{3})+i\omega b_{13}(\vec{u}_{3}-\vec{u}_{1}). \end{matrix}$ (A46)

Any vector field can be decomposed into a sum of a P-wave field and two S-wave fields. If the *z*-axis is the specified direction in space, we have

(A47)

Substituting Eq. (A47) into (A44), (A45) and (A46), we can obtain (where the term $\chi$ is 0, which is because there is no SH wave):

(A48)

(A49)

(A50)

Values in gradient and curl in Eqs (A48), (A49) and (A50) are zero. Thus, there are six equations, which can be divided into two coupled equations. The first group is the gradient of the three equations, which is the compressional wave potential, and the value of this part is zero. Besides, $\nabla^{2}\text{=}-k_{\text{P}}^{2}$, the first group can be written in the matrix form as

(A51)

in Eq. (A51) has a non-zero solution, the determinant of the left matrix should be zero. It is a cubic polynomial of $k_{\text{P}}^{2}$, which has three roots: $k_{\text{P1}}^{2}$, $k_{\text{P2}}^{2}$ and $k_{\text{P3}}^{2}$. Take the real part of three wavenumbers as positive, and the real part of $k_{\text{P1}}$ is less than that of $k_{\text{P3}}$. The corresponding three $k_{\text{P}}^{2}$ can get three $l_{1}$ and three $l_{2}$, which is given by $l _{11}$, $l_{12}$, $l_{13}$ and $l_{21}$, $l_{22}$ and $l_{23}$, corresponding to three kinds of compressional waves.

The second group is the curl of the three equations, which is the shear wave potential, and the value of this part is zero. Besides, $\nabla^{2}\text{=}-k_{\text{S}}^{2}$, the second group can be written in the matrix form as

$\left[\begin{matrix} \begin{matrix} -(\mu_{1}+\frac{1}{2}\mu_{13})k_{\text{S}}^{2} \\ +(\rho_{11}+\rho_{12}+\rho_{13})\omega^{2} \end{matrix} & (\rho_{11}+\rho_{12}+\rho_{13})\omega^{2} & \begin{matrix} -(\mu_{3}+\frac{1}{2}\mu_{13})k_{\text{S}}^{2} \\ +(\rho_{11}+\rho_{12}+\rho_{13})\omega^{2} \end{matrix} \\ \rho_{12}\omega^{2}-i\omega b_{12} & \rho_{22}\omega^{2}+i\omega(b_{12}+b_{23}) & \rho_{23}\omega^{2}-i\omega b_{23} \\ \begin{matrix} -\frac{1}{2}\mu_{13}k_{\text{S}}^{2} \\ +\rho_{13}\omega^{2}-i\omega b_{13} \end{matrix} & \rho_{23}\omega^{2}-i\omega b_{23} & \begin{matrix} -\mu_{3}k_{\text{S}}^{2}\rho_{33}\omega^{2} \\ +i\omega(b_{23}+b_{13}) \end{matrix} \end{matrix}\right]\left[\begin{matrix} \eta_{1} \\ l_{3}\eta_{1} \\ \frac{l_{3}}{l_{4}}\eta_{1} \end{matrix}\right]=0.$ (A52)

Let the determinant of the left matrix be zero, we can obtain two values of $k_{\text{S}}^{2}$, Substitute $k_{\text{S}}^{2}$ into Eq. (A52), two $l_{3}$ and two $l_{4}$ can be obtained, which can be expressed as $l_{31}$, $l_{32}$, $l_{41}$, and $l_{42}$, corresponding to two kinds of shear waves.
